# The complete mitochondrial genome of *Homatula berezowskii* (Cypriniformes, Nemacheilidae, Nemachilinae)

**DOI:** 10.1080/23802359.2020.1829517

**Published:** 2020-10-21

**Authors:** Yang Li, Haolin Mo, Jing Xu, Lian Wu, Lixin Wang

**Affiliations:** a College of Animal Science and Technology, Northwest A&F University, Yangling, China; b Key Laboratory of Molecular Biology for Agriculture, Northwest A&F University, Yangling, China; c Department of Biotechnology, Xi'an University, Xi’an, China

**Keywords:** *Homatula berezowskii*, complete mitochondrial genome, taxonomic status

## Abstract

*Homatula berezowskii* (Günther 1896), used to be recognized as a synonym of *Homatula variegate*, was now identified as a valid species. However, these morphological studies lack genetic evidence to support. In the present study, we determined the first complete mitochondrial genome of *H. berezowskii* by Sanger dideoxy sequencing. The genome size is 16,570 bp and it contains 13 protein-coding genes, 22 transfer RNA genes, 2 ribosomal RNA genes, and a non-coding control region (D-loop). Phylogenetic analyses based on the complete mitochondrial genome indicated the *H. berezowskii* was clustered with *H. potanini* first and then with the *H. variegate*. This work may be helpful to clarify the taxonomic status of *H. berezowskii*.

*Homatula berezowskii* (Günther 1896), an endemic freshwater fish to China, is only distributed in the upstream of the Yellow River and the Yangtze River, and their tributaries (Ding 1994). This fish used to be considered as a synonym of *H. variegate*was recently identified as a valid species (Hu and Zhang 2010; Gu and Zhang 2012). However, most of the taxonomic studies of this fish were based on the morphological parameters, only a partial sequence of cytochrome b (cytb) was used to revise the classification of *Homatula* and *Schistura* in the Sichuan province of China (Xiong et al. 2017). To further clarify the taxonomic status of *H. berezowskii*, we sequenced its complete mitochondrial genome.

The *Homatula berezowskii* sample of *H. berezowskii* was collected from Qujing City (103.7°E, 25.6°N), Yunnan Province, China, and now is stored in Key Laboratory of Molecular Biology for Agriculture, Northwest A&F University, Yangling, China (voucher no. FS-2014-Y03). Total DNA was extracted by using the Animal Genomic DNA extraction Kit (Tiangen Biotech, Beijing, China). The whole mitochondrial genome was PCR amplified, Sanger-sequenced, and assembled following the method described by Zhou et al. (2014).

The complete *H. berezowskii* mitochondrial genome (GenBank accession no. NC_040302.1) was 16,570 bp in length, including 13 protein-coding genes (PCGs), 22 tRNA genes, 2 rRNA genes, and a non-coding control region (D-loop region). All the PCGs employ ATG as the start codon, except for COX1, using the start codon GTG. As for the termination codon, six genes (ND1, ND2, COX1, ND3, ND4, ND6) use TAG, and four genes (ATP8, ATP6, ND4L, ND5) use TAA as their stop codon. The remaining three PCGs use the incomplete stop codon T–. No TA– stop codon, which differs from *Homatula potanini* (Que et al. 2012) and *Homatula variegatus* (Shi et al. 2014).

The phylogenetic tree was constructed with 16 fish species of the Nemachilinae subfamily by using the maximum likelihood (ML) method with 1000 bootstrap replicates. Results showed that *H. berezowskii*is sister to *H. potanini*, and shares a recent common ancestor with *H. variegatus* (Figure 1). The genetic data agree with the morphological data (Xiong et al. 2017). The *Schistura* spp., *Triplophysa* spp., and *Lefua* spp. were also clustered together, but *Micronemacheilus* spp. and *Oreonectes* spp. were intermingled with other species, suggesting further taxonomic investigations are needed.

**Figure 1.**
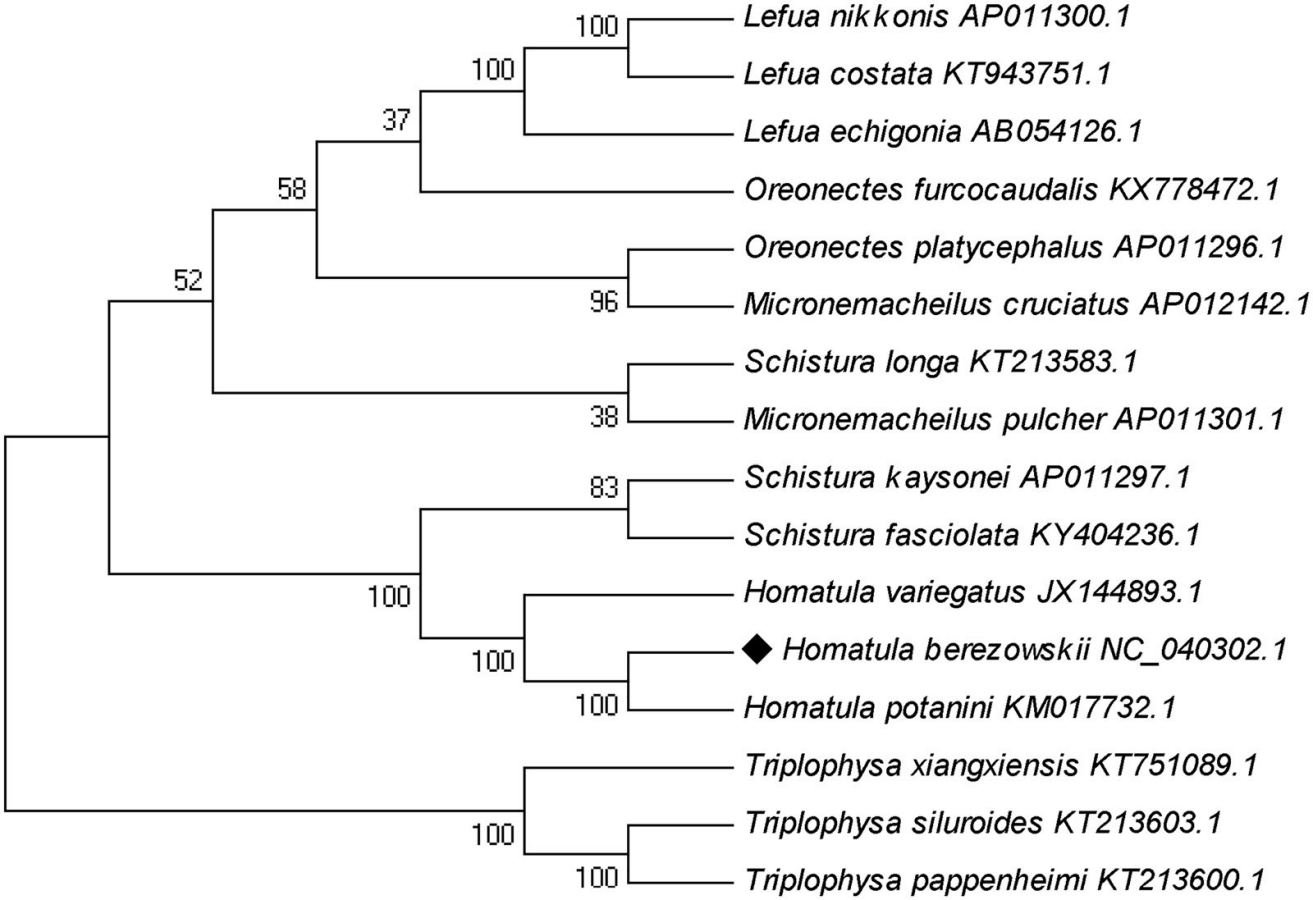
The phylogenetic tree of 16 Nemachilinae based on ML (1000 bootstrap replicates).

## Data Availability

The data that support the findings of this study are openly available in NCBI at https://www.ncbi.nlm.nih.gov/, reference number NC_040302.1.
